# Workplace violence, occupational burnout, and post-traumatic stress among healthcare workers in Armenia

**DOI:** 10.3389/fpubh.2026.1926848

**Published:** 2026-08-19

**Authors:** Meri Mkhitaryan, Armine Chopikyan, Armine Harutyunyan, Tatevik Arakelyan, Armen Muradyan, Artashes Tadevosyan

**Affiliations:** 1 Yerevan State Medical University Named after Mkhitar Heratsi, Yerevan, Armenia; 2 General Education, American University of Armenia, Yerevan, Armenia

**Keywords:** Armenia, healthcare workers, mental health, occupational burnout, post-traumatic stress disorder (PTSD), workplace violence

## Abstract

**Background:**

Workplace violence is a significant occupational hazard in healthcare systems worldwide and may have serious psychological consequences for healthcare workers. Exposure to violence can contribute to the development of occupational burnout and post-traumatic stress disorder (PTSD), negatively affecting both staff well-being and the quality of healthcare services.

**Aim:**

To analyze the types of workplace violence, associated risk factors, and the relationship between exposure to violence, occupational burnout, and post-traumatic stress disorder among healthcare workers.

**Methods:**

A quantitative cross-sectional non-experimental study was conducted among 432 healthcare workers from Yerevan and the regions of Armenia. Workplace violence was assessed using the collaboratively developed and internationally validated questionnaire within ICREATE project. Burnout was evaluated using the Maslach Burnout Inventory, while PTSD symptoms were assessed using the DSM-5 PCL-5 questionnaire among participants who reported exposure to violence (n = 305). Statistical analyses included Chi-square tests, Cramer’s V coefficient, independent samples t-tests, one-way ANOVA, and logistic regression.

**Results:**

Verbal violence was reported by 333 participants, physical violence by 184, sexual harassment by 77 and sexual violence by 10 healthcare workers. High or very high levels of occupational burnout were observed in the majority of respondents. Statistical analysis revealed significant associations between physical and verbal violence and higher levels of emotional exhaustion, depersonalization, and overall burnout (*p* < 0.05). Ordinal logistic regression analysis indicated that healthcare workers exposed to physical violence had approximately three times higher odds of experiencing higher levels of burnout (OR≈3.02; 95% CI: 1.98–4.63). Among participants exposed to violence, 64.9% reached the threshold score for probable PTSD. Physical violence was significantly associated with PTSD (χ^2^ = 30.130, *p* = 0.000), and logistic regression analysis showed that exposure to physical violence increased the likelihood of PTSD by approximately 3.4 times.

**Conclusion:**

Workplace violence is strongly associated with occupational burnout and PTSD among healthcare workers. Physical and verbal violence represent key predictors of adverse psychological outcomes.

## Introduction

The healthcare system is a complex sector characterized by hierarchical and bureaucratic structures, where professional activities are carried out under conditions of high responsibility, time constraints, and relative scarcity of resources. In such an environment, any additional stressor particularly violence may act as a powerful maladaptive factor, affecting the mental health and professional performance of healthcare workers (1–9).

The healthcare sector is the most common area of workplace violence. A considerable proportion of healthcare workers experience verbal, psychological, or physical aggression during their professional careers. The primary sources of violence are patients and their relatives․ The highest levels of risk are observed in emergency departments, admission units, surgical and intensive care units. During the pandemic, the frequency of violence increased due to public fear, the overload of the healthcare system, and the limited accessibility of healthcare services (10–15).

Violence includes not only acute incidents but also repeatedly occurring minor episodes that lead to chronic stress. Recurrent aggression, threats, and feelings of insecurity create psychological tension that contributes to the development of occupational burnout syndrome. The concept of burnout was theoretically formulated by Freudenberger and later systematized through Maslach’s three-component model, which includes emotional exhaustion, depersonalization, and reduced personal accomplishment (16).

Emotional exhaustion is considered the core component and is manifested through depletion of energy, chronic fatigue, anxiety, and the predominance of negative emotions. Depersonalization is expressed through social withdrawal and a detached attitude toward the profession, while reduced personal accomplishment is accompanied by decreased self-esteem and reduced effectiveness of professional activity (17, 18).

According to international data, the prevalence of burnout among healthcare workers in Europe ranged between 29–57.6%, while during the COVID-19 pandemic it reached up to 69%. In radiology departments in Germany, burnout levels reached up to 76.7%, in China 72.9%, and in Indonesia 37.5%, while in Southeast Asian countries the overall prevalence ranged from 26.8 to 90.0%, including both mild and severe forms. At the same time, anxiety was reported in 44.6% of healthcare workers in China, and depressive symptoms in 50.4%, both of which are closely associated with higher levels of burnout. Among healthcare workers exposed to violence, burnout syndrome develops more rapidly and tends to have a more severe course. Violence disrupts the sense of professional identity, reduces the perceived meaning of work, and deepens psychological maladaptation (2–9, 19).

Acute or recurrent episodes of violence may also lead to the development of post-traumatic stress disorder (PTSD). According to the DSM-5 classification, PTSD is characterized by a combination of intrusion symptoms (recurrent memories and dreams), avoidance, negative cognitive and emotional changes, and hyperarousal symptoms (20). In the general adult population, the prevalence of PTSD is estimated to range from 1.9 to 8.8%; however, among healthcare workers exposed to violence it is considerably higher, with some studies reporting symptoms consistent with the clinical criteria for PTSD in up to one third of cases (21).

PTSD has not only psychological but also somatic consequences, including an increased risk of cardiovascular diseases, gastrointestinal disorders, and weakening of the immune system. Chronic hyperarousal and sleep disturbances reduce work concentration and the quality of decision-making, thereby increasing the likelihood of professional errors. In addition, PTSD may contribute to an increased risk of depression, anxiety disorders, and substance use (15, 22, 23).

Thus, violence in the healthcare system represents a multi-level risk factor contributing to the development of both occupational burnout syndrome and post-traumatic stress disorder. The problem has not only individual but also systemic consequences, affecting workforce stability, team dynamics, and the quality of healthcare services (24, 25). From a public health perspective, it is necessary to develop comprehensive strategies for violence prevention, early detection, and psychological support aimed at protecting the mental health of healthcare workers and ensuring the sustainability of healthcare systems.

Numerous international studies have documented the widespread prevalence of workplace violence against healthcare workers and its association with the development of occupational burnout and post-traumatic stress disorder (PTSD). However, the majority of published research has been conducted in healthcare systems of high-income, developed countries. In contrast, the prevalence, characteristics, and consequences of workplace violence remain insufficiently investigated in low- and middle-income countries, including Armenia (26).

Although the international scientific literature has made a substantial contribution to understanding the causes and consequences of violence against healthcare workers, these findings cannot be directly generalized to the Armenian healthcare system. Differences in healthcare system organization, as well as socio-cultural and contextual factors, may influence both the manifestations of workplace violence and its psychological and occupational consequences (26, 27).

In Armenia, incidents of violence against healthcare workers are periodically reported in the mass media. Nevertheless, statistical data and systematic scientific evidence regarding the prevalence, forms, and risk factors of workplace violence, as well as its association with occupational burnout and post-traumatic stress disorder among healthcare workers, remain largely unavailable.

This study aims to address this important knowledge gap by providing the first comprehensive assessment of workplace violence against healthcare workers in Armenia. Specifically, it investigates the prevalence and major forms of workplace violence, identifies associated risk factors, development of occupational burnout and post-traumatic stress disorder. The findings of this study will provide evidence to support the development of evidence-based preventive interventions and policies aimed at improving the safety and protection of healthcare workers within the Armenian healthcare system.

## Aim

To analyze the types of workplace violence, risk factors and the association between violence exposure, burnout, and post-traumatic stress disorder among healthcare workers.

## Methods

### Study design and participants

A quantitative cross-sectional study was conducted among healthcare workers employed in healthcare facilities across Yerevan and the regions of Armenia. The study aimed to examine the prevalence of workplace violence and its association with occupational burnout and post-traumatic stress disorder (PTSD).

The minimum required sample size for the quantitative study was calculated using the formula commonly applied for cross-sectional studies:


$$n=z^{2}\times p\times q/d^{2}$$

where n represents the required sample size; z is the z-score corresponding to a 95% confidence level and equals 1.96; p (prevalence) represents the expected prevalence of the studied phenomenon, which was selected considering the maximum uncertainty level and was set at 0.5; q = 1 − p, representing the absence of the phenomenon, which was also equal to 0.5 in this study; and d represents the acceptable margin of error, set at 5% (0.05). Based on this calculation, the minimum required sample size was *n* = 384. To increase statistical power and improve representativeness, a total of 432 healthcare workers were ultimately enrolled in the study. The healthcare workers participating in the study were selected based on the principle of availability, among the medical staff working during visits to medical facilities.

Participants included physicians, resident doctors, nurses, midwives, and other healthcare professionals working in different healthcare settings. Both frontline personnel, including emergency department, ambulance, and intensive care unit staff, and healthcare workers from other clinical departments were eligible to participate. Healthcare workers were eligible for inclusion if they had at least 2 years of professional work experience.

### Data collection and study instruments

Data were collected using a structured questionnaire package consisting of three validated instruments.

Workplace violence was assessed using the questionnaire developed within the ICREATE project, an international collaborative initiative focused on violence against healthcare providers (28). The instrument has been used across several countries and captures different forms of workplace violence, including physical violence, verbal abuse, sexual harassment, sexual violence, and theft. Additional questions explored the circumstances of violent incidents, including the timing and location of the event, characteristics of perpetrators, reporting behaviors, witnessed incidents, and perceived consequences on professional performance and well-being.

Occupational burnout was evaluated using the Maslach Burnout Inventory (MBI), one of the most widely used instruments for measuring burnout among healthcare professionals (18, 29). The questionnaire consists of 22 items and assesses three core dimensions of burnout: emotional exhaustion, depersonalization, and personal accomplishment. Based on established scoring criteria, participants were categorized into burnout severity groups ranging from low to very high levels. The emotional exhaustion component was assessed based on items 1, 2, 3, 6, 8, 13, 14, 16, and 20 of the questionnaire (maximum score = 54).

Depersonalization was assessed based on items 5, 10, 11, 15, and 22 (maximum score = 30).

Reduced personal accomplishment was assessed using items 4, 7, 9, 12, 17, 18, 19, and 21 (maximum score = 48).

The overall burnout level was assessed based on all 22 items of the questionnaire. The psychological impact of workplace violence was further assessed using the PTSD Checklist for DSM-5 (PCL-5), a validated self-report instrument designed to evaluate symptoms of post-traumatic stress disorder according to DSM-5 diagnostic criteria (30). The questionnaire includes 20 items covering four symptom clusters: intrusion, avoidance, negative alterations in cognition and mood, and hyperarousal. The PCL-5 was administered only to participants who reported experiencing at least one violent incident during their professional activities.

The internal consistency of the study instruments was evaluated in the current sample using Cronbach’s alpha coefficients. The Maslach Burnout Inventory (MBI) and the PTSD Checklist for DSM-5 (PCL-5) demonstrated satisfactory internal consistency, supporting the reliability of these instruments for assessing occupational burnout and PTSD symptoms among Armenian healthcare workers. The MBI demonstrated excellent internal consistency (Cronbach’s *α* = 0.89), while the PCL-5 also showed excellent reliability (Cronbach’s α = 0.91).

### Statistical analysis

Data were coded, cleaned, and analyzed using IBM SPSS Statistics version 26. Descriptive statistics were used to summarize participant characteristics and study variables. Categorical variables were presented as frequencies and percentages.

Associations between categorical variables were examined using Pearson’s chi-square test, while Cramer’s V coefficient was calculated to assess the strength of statistically significant associations. Independent-samples t-tests and one-way analysis of variance (ANOVA) were applied to compare mean burnout and PTSD scores across study groups.

Prior to inferential analyses, the assumptions underlying the statistical methods were evaluated. Normality of continuous variables was assessed using the Shapiro–Wilk test together with visual inspection of histograms and Q–Q plots. Homogeneity of variances for independent-samples t-tests and one-way ANOVA was evaluated using Levene’s test. For regression analyses, multicollinearity among predictor variables was assessed using variance inflation factors (VIFs), with values indicating no evidence of problematic multicollinearity. The goodness-of-fit of the binary logistic regression model was evaluated using the Hosmer–Lemeshow test. For the ordinal logistic regression model, the proportional odds assumption was assessed using the Test of Parallel Lines, while overall model fit was evaluated using the Model Fitting Information statistics. Statistical significance was defined as a two-sided *p*-value < 0.05.

### Ethical considerations

Participation in the study was voluntary and anonymous. Prior to data collection, participants were informed about the objectives of the study and provided informed consent. Confidentiality and privacy were ensured throughout the research process.

The study protocol was reviewed and approved by the Ethics Committee of Yerevan State Medical University (protocol #4/2, 11.25.2021).

## Results

### Descriptive statistics

A total of 432 healthcare workers participated in the study (Table 1).

**Table 1:** Sociodemographic and professional characteristics of the study participants (*N* = 432).

Variable	Category	*n*	%
Sex	Male	73	16.9
	Female	359	83.1
Age group (years)	20–24	13	3.0
	25–29	62	14.4
	30–34	57	13.2
	35–39	46	10.6
	40–44	72	16.7
	45–49	41	9.5
	50–54	50	11.6
	55–59	34	7.9
	≥60	57	13.2
Profession	Nurses	190	44.0
	Physicians	143	33.1
	Resident doctors	24	5.6
	Other	75	17.4
Work experience	2–5 years	127	29.4
	6–10 years	65	15.0
	>10 years	240	55.6

Regarding professional composition, among the “other specialties” category the most frequently represented group was midwives (*n* = 72; 16.7%). Additionally, two radiology technicians and one physician-administrator were included in this category.

With respect to professional experience, 240 participants (55.6%) had more than 10 years of work experience, 65 participants (15.0%) had 6–10 years of experience, and 127 participants (29.4%) had 2–5 years of professional experience.

### Circumstances and types of violent incidents

Physical assault was reported by 184 participants, verbal violence by 333 participants, sexual violence by 10 healthcare workers, and sexual harassment by 77 participants.

Among the 323 healthcare workers who reported experiencing violence, 133 (41.4%) indicated that the incident occurred during the day shift, while 188 (58.6%) reported that the incident occurred during the night shift. Two respondents did not answer this question.

Regarding the day of occurrence 211 participants (66.0%) reported that the incident occurred on a working day, while 108 participants (33.9%) indicated that it occurred on a non-working day (weekend, holidays). Four respondents did not answer this question.

### Assessment of occupational burnout syndrome

Occupational burnout resulting from violence or attacks against healthcare workers was assessed using the Maslach Burnout Inventory. Three main components of burnout were evaluated: emotional exhaustion, depersonalization, and reduced personal accomplishment (reduced professional motivation), as well as the overall level of burnout.

High and very high levels of emotional exhaustion were observed in 63.2% of participants (*n* = 273) (Table 2).

**Table 2:** Distribution of occupational burnout levels by MBI dimensions (*N* = 432).

Burnout dimension	Level	*n*	%
Emotional exhaustion	Low	31	7.2
	Moderate	128	29.6
	High	171	39.6
	Very high	102	23.6
Depersonalization	Very low	2	0.5
	Low	85	19.7
	Moderate	112	25.9
	High	150	34.7
	Very high	83	19.2
Reduced personal accomplishment	Very low (extremely reduced motivation)	216	50.0
	Low	186	43.1
	Moderate	30	6.9
	High	0	0.0
	Very high	0	0.0
Overall burnout	Low	1	0.2
	Moderate	54	12.5
	High	291	67.4
	Very high	86	19.9

Regarding depersonalization, high and very high levels were identified in 53.9% of respondents (*n* = 233). Low or very low depersonalization was observed in 20.2% (*n* = 87) of participants.

In contrast, the dimension of reduced personal accomplishment showed a markedly unfavorable distribution, with extremely low or low levels of professional motivation reported by 93.1% of healthcare workers (*n* = 402), while higher levels were not observed.

Overall, occupational burnout levels were predominantly elevated, with 87.3% of participants (*n* = 377) classified as having high or very high burnout.

### Emotional exhaustion and violence

Independent t-test results showed statistically significant differences among emotional exhaustion scores among healthcare workers exposed to physical violence (*p* = 0.000) and verbal violence (*p* = 0.001).

No statistically significant association was found with sexual violence (*p* = 0.29) and sexual harassment (*p* = 0.09).

No statistically significant differences were observed with respect to sex, profession, or age (*p* > 0.05).

### Depersonalization and violence

Statistically significant differences in depersonalization scores were observed for physical violence (*p* = 0.000) and verbal violence (*p* = 0.001).

No significant association was found with sexual violence or sexual harassment (*p* > 0.05).

While no association was found with sex or profession. Statistically significant difference was observed between depersonalization scores and age (*p* = 0.008).

#### Reduced professional motivation and violence

Significant differences were found between reduced professional motivation and physical violence and verbal violence (*p* < 0.05).

No statistically significant associations were observed with sexual violence, sexual harassment, sex, profession, or age (*p* > 0.05).

#### Overall burnout and violence

Analysis of overall burnout levels demonstrated significantly higher burnout scores among healthcare workers exposed to physical violence (*p* = 0.001) and verbal violence (*p* = 0.01).

No statistically significant associations were observed with sexual violence, sexual harassment, sex, profession, or age (*p* > 0.05).

Associations between burnout components and sociodemographic variables, forms of violence, and work shifts were analyzed using the Chi-square test (Table 3).

**Table 3:** Associations between workplace violence, sociodemographic factors, and burnout dimensions (Chi-square analysis).

Burnout dimension	Variable	χ^2^	*p*-value	Cramer’s V	Strength of association
Emotional exhaustion	Physical violence	33.814	<0.001	0.280	Moderate
	Verbal violence	9.500	0.023	0.149	Weak
	Work shift	10.381	0.016	0.180	Weak
	Sex	NS	>0.05	-	Not significant
	Age	NS	>0.05	-	Not significant
	Profession	NS	>0.05	-	Not significant
	Sexual harassment	NS	>0.05	-	Not significant
	Sexual violence	NS	>0.05	-	Not significant
Depersonalization	Physical violence	33.793	<0.001	0.280	Moderate
	Verbal violence	12.948	0.012	0.170	Weak
	Age	Significant	0.008	-	Weak–moderate
	Sex	NS	>0.05	-	Not significant
	Profession	NS	>0.05	-	Not significant
Reduced professional accomplishment	Work shift	7.600	0.022	0.154	Weak
	Physical violence	NS	>0.05	-	Not significant
	Verbal violence	NS	>0.05	-	Not significant
	Sex	NS	>0.05	-	Not significant
	Age	NS	>0.05	-	Not significant
Overall burnout	Physical violence	30.994	<0.001	0.268	Moderate
	Verbal violence	NS	>0.05	-	Not significant
	Sexual harassment	NS	>0.05	-	Not significant
	Sexual violence	NS	>0.05	-	Not significant
	Sex	NS	>0.05	-	Not significant
	Age	NS	>0.05	-	Not significant
	Profession	NS	>0.05	-	Not significant

Emotional exhaustion showed statistically significant associations with physical violence (χ^2^ = 33.814, *p* = 0.000, Cramer’s V = 0.28), verbal violence (χ^2^ = 9.5, *p* = 0.023, Cramer’s V = 0.149) and work shift (χ^2^ = 10.381, *p* = 0.016, Cramer’s V = 0.18).

Depersonalization showed significant associations with physical assault (χ^2^ = 33.793, *p* = 0.000, Cramer’s V = 0.28) and verbal violence (χ^2^ = 12.948, *p* = 0.012, Cramer’s V = 0.17).

Reduced professional motivation showed a significant association was observed with work shift (χ^2^ = 7.6, *p* = 0.022, Cramer’s V = 0.154), indicating a relationship between lower professional motivation and night shifts.

Overall burnout showed a statistically significant association only with physical violence (χ^2^ = 30.994, *p* = 0.000, Cramer’s V = 0.268).

Ordinal logistic regression analysis was performed using burnout level (low, moderate, high, very high) as the dependent variable and exposure to physical violence (yes/no) as the independent variable (Table 4).

**Table 4:** Association between physical violence and burnout (ordinal logistic regression).

Physical violence and burnout	Estimate(B)	OR	*p*-value	*p*-value (test of parallel lines)
	1.107	3.02	0.000	0.130

Healthcare workers exposed to physical violence were approximately three times more likely to have higher burnout levels compared to those not exposed to violence (OR ≈ 3.02; 95% CI: 1.98–4.63; *p* = 0.000).

The odds ratio was calculated using the formula: OR = e^B, where e is the base of the natural logarithm (≈ 2.718), and B is the regression coefficient (B = 1.107).

The Test of Parallel Lines (*p* > 0.05) indicated that the proportional odds assumption was satisfied. Model Fitting Information showed statistical significance (*p* = 0.000), confirming the adequacy of the model.

The analysis confirms that physical violence is an independent predictor significantly increasing the probability of higher burnout levels among healthcare workers.

### Assessment of post-traumatic stress disorder (PTSD)

The PTSD Checklist for DSM-5 (PCL-5) was completed by 305 of the 432 study participants. PTSD symptom clusters were assessed according to DSM-5 criteria, including intrusion (Cluster B), avoidance (Cluster C), negative alterations in cognition and mood (Cluster D), and hyperarousal (Cluster E).

Cluster B scores ≥2 were observed in 296 participants, cluster C ≥ 2 in 264 participants, and clusters D and E ≥ 2 in all 305 participants, indicating a high prevalence of probable PTSD symptoms.

Analysis of the total PCL-5 scores showed that the threshold for probable PTSD diagnosis (≥31–33 points) was reached in 198 healthcare workers (64.9%).

Statistical analysis showed that PTSD scores were significantly higher among healthcare workers exposed to physical violence (*p* = 0.000) and verbal violence (*p* = 0.001).

No statistically significant differences were found with respect to sex, sexual violence, or sexual harassment (*p* > 0.05). One-way ANOVA analysis also showed no significant association between PTSD levels and age or profession (*p* > 0.05).

Chi-square analysis revealed a statistically significant association between physical violence and PTSD (χ^2^ = 30.130, *p* = 0.000, Cramer’s V = 0.264), indicating a moderate association.

Binary Logistic regression analysis demonstrated that PTSD was 3.4 times more likely to develop among healthcare workers exposed to physical violence compared to those not exposed (OR = 3.447; 95% CI: 2.124–5.596; *p* = 0.000).

## Discussion

The findings of the present study demonstrate that workplace violence is highly prevalent among healthcare workers in Armenia and is strongly associated with both occupational burnout and post-traumatic stress disorder (PTSD). Verbal violence was the most frequently reported form of violence, followed by physical violence, while sexual harassment and sexual violence were reported less frequently. These findings are consistent with international evidence indicating that verbal aggression is the most common form of workplace violence in healthcare settings worldwide (13, 16, 31).

The study sample was predominantly female, reflecting the global trend in the health and care sector, where women constitute approximately 70% of the workforce (15). This distribution is consistent with the gender composition of the healthcare workforce worldwide․

The prevalence of occupational burnout observed in the current study was remarkably high. More than two-thirds of participants demonstrated high overall burnout levels, while approximately one-fifth experienced very high burnout. Similarly, elevated burnout levels have been reported among healthcare workers during and after the COVID-19 pandemic. A systematic review conducted by Hiver et al. (4) reported burnout prevalence estimates ranging from 29 to 58% among European physicians, while studies from Poland, China, and Indonesia identified substantial increases in burnout associated with excessive workload and psychological distress (5–7, 32). The prevalence observed in the present study appears to be higher than many international estimates, suggesting that Armenian healthcare workers may be exposed to a combination of occupational stressors that intensify emotional exhaustion and depersonalization.

Physical and verbal violence were significantly associated with emotional exhaustion, depersonalization, and overall burnout. These findings are consistent with previous studies demonstrating that exposure to aggression from patients and their relatives contributes directly to chronic workplace stress and emotional depletion (3, 17). Violence may undermine healthcare workers’ sense of safety and professional identity, ultimately leading to cynicism, reduced empathy, and emotional distancing from patients. Such mechanisms correspond closely with Maslach’s theoretical framework, which identifies emotional exhaustion and depersonalization as central dimensions of burnout (18).

The ordinal logistic regression analysis further demonstrated that healthcare workers exposed to physical violence had approximately three times higher odds of experiencing more severe burnout. Similar associations have been reported in several international investigations. For example, Giménez Lozano et al. (17) concluded that workplace violence is one of the strongest occupational predictors of burnout among physicians and nurses. The consistency of these findings across different healthcare systems suggests that violence represents a universal occupational hazard with profound psychological consequences.

An important finding of this study is the exceptionally high prevalence of probable PTSD among healthcare workers exposed to violence. Nearly two-thirds of participants exceeded the recommended PCL-5 threshold for probable PTSD. This prevalence substantially exceeds estimates reported in several international meta-analyses. A systematic review examining healthcare workers after infectious disease outbreaks reported pooled PTSD prevalence estimates generally ranging between 20 and 35% (21). Similarly, Liu et al. (24) identified significantly lower pooled prevalence rates among healthcare workers during the COVID-19 pandemic. The higher prevalence observed in the current study may reflect cumulative exposure to repeated violent incidents, limited access to psychological support services, or underdeveloped institutional mechanisms for violence prevention and response.

The present study also demonstrated a significant relationship between physical violence and PTSD. Healthcare workers exposed to physical violence were more than three times more likely to meet criteria for probable PTSD than those not exposed. These findings are consistent with established trauma literature indicating that direct exposure to threats, aggression, and physical assault increases the risk of intrusive memories, avoidance behaviors, emotional dysregulation, and hyper arousal symptoms (20, 24). Repeated exposure to violence may further reinforce traumatic stress responses through cumulative psychological burden.

Interestingly, no statistically significant associations were identified between burnout or PTSD and demographic variables such as sex, age, or professional category. Similar findings have been reported by several recent studies suggesting that occupational exposures may exert a stronger influence on psychological outcomes than individual demographic characteristics (8, 19). This observation highlights the importance of organizational factors and workplace conditions in determining healthcare workers’ mental well-being.

From a public health perspective, the findings emphasize the urgent need for comprehensive workplace violence prevention strategies within healthcare institutions. International organizations, including the World Health Organization (15), have repeatedly highlighted the importance of violence prevention policies, staff training, reporting systems, and access to psychological support services. Strengthening institutional responses to workplace violence may reduce burnout, improve mental health outcomes, enhance staff retention, and ultimately contribute to better quality of patient care.

The findings of the present study also have important practical implications for healthcare management and occupational health policy. Hospital administrators should prioritize the development of comprehensive workplace violence prevention strategies, including clear reporting procedures, staff training in conflict de-escalation, improved security measures, and institutional zero-tolerance policies toward violence. Regular psychological screening for burnout and PTSD, together with timely access to counseling and employee support services, may facilitate early identification of healthcare workers at risk and reduce the long-term psychological consequences of workplace violence. At the policy level, integrating violence prevention into occupational health programmes may contribute to improving workforce well-being, staff retention, and the overall quality and safety of healthcare services.

The present study has several limitations that should be considered when interpreting the findings. First, the cross-sectional design precludes establishing causal relationships between workplace violence, occupational burnout, and PTSD. Second, convenience sampling may limit the generalizability of the findings to all healthcare workers in Armenia. Third, all data were collected using self-reported questionnaires and are therefore subject to information bias, including recall bias and social desirability bias. Participants may have unintentionally misreported their experiences or psychological symptoms. Furthermore, workplace violence may have been under-reported because some healthcare workers may perceive violence as an unavoidable part of their professional duties, fear negative professional consequences, or be reluctant to disclose such experiences. Consequently, the true prevalence of workplace violence may be underestimated. Despite these limitations, this study represents one of the first comprehensive investigations in Armenia examining the relationships between workplace violence, occupational burnout, and PTSD among healthcare workers and provides important evidence to support future research and policy development.

## Conclusion

Exposure to workplace violence contributes directly to the development of occupational burnout among healthcare workers.

High levels of burnout are more frequently observed among healthcare workers exposed to physical and verbal violence, particularly manifested as emotional exhaustion and depersonalization.

Reduced professional motivation is more commonly associated with night shift work.

Overall, the primary predictors of burnout are related to stressful occupational environmental factors rather than sociodemographic characteristics.

Physical and verbal violence significantly increase the risk of developing post-traumatic stress disorder (PTSD).

Workplace violence directly contributes to occupational burnout among healthcare workers.

These findings emphasize the combined impact of workplace stressors and violence exposure, highlighting the need for integrated organizational and psychological interventions.

## Recommendations

Enhancing workplace security and safety: implementation of alarm systems, involvement of trained security personnel and strengthening of existing security services.
Organizational and legal measures: development and implementation of workplace violence prevention policies and establishment of institutional committees responsible for monitoring and managing violence-related incidents.
Psychological support for healthcare workers: provision of psychological counseling services for healthcare workers within healthcare facilities, early screening and identification of PTSD symptoms and occupational burnout and implementation of psychological support programs.
Educational and awareness-raising programs: training healthcare workers in violence prevention, conflict management, and professional rights, alongside promoting public awareness of respectful interactions with healthcare professionals.
Cultural and workplace reforms: Cultural and workplace reforms: fostering a zero-tolerance culture toward violence against healthcare workers and optimizing work schedules and workload distribution.

## Data Availability

The raw data supporting the conclusions of this article will be made available by the authors, without undue reservation.
